# Selective Laser Trabeculoplasty for Glaucoma Secondary to Emulsified Silicone Oil after Pars Plana Vitrectomy: A Pilot Study

**DOI:** 10.1155/2014/469163

**Published:** 2014-04-13

**Authors:** Zeynep Alkin, Banu Satana, Abdullah Ozkaya, Berna Basarir, Cigdem Altan, Ahmet Taylan Yazici, Ahmet Demirok

**Affiliations:** ^1^Beyoglu Eye Training and Research Hospital, Bereketzade Cami Sokak, No. 2, Beyoglu, 34421 Istanbul, Turkey; ^2^Medeniyet University, Dumlupinar Mahallesi, D-100 Karayolu, No. 98, Kadikoy, 34270 Istanbul, Turkey

## Abstract

*Background*. To investigate the efficacy of selective laser trabeculoplasty (SLT) for lowering intraocular pressure (IOP) in patients with open angle glaucoma (OAG) secondary to emulsified silicone oil (SO). *Methodology/Principal Findings*. Prospective, interventional, consecutive case series of 11 eyes with sustained elevation of IOP after SO removal. The mean IOP at baseline, week 1, month 1, month 3, and month 6 was evaluated. The mean baseline IOP was significantly decreased from 25 ± 2.7 mmHg to 18.4 ± 5.5 mmHg at week 1 (*P* = 0.01), 17.9 ± 3.1 mmHg at month 1 (*P* = 0.008), 15.8 ± 3.9 mmHg at month 3 (*P* = 0.003), and 16.2 ± 4.7 mmHg at month 6 (*P* = 0.004). IOP < 21 mmHg was achieved in 91% of the eyes without a significant complication at month 6. *Conclusion/Significance*. SLT may be successful for lowering IOP in patients with OAG secondary to emulsified SO which was not controlled with maximum antiglaucomatous medical treatment.

## 1. Introduction

Silicone oil (SO) as an endotamponade in the treatment of retinal detachments was first introduced by Cibis et al. in 1962 [1]. Along with the rapid development of complicated posterior segment surgeries, the use of SO has greatly increased [2]. Although SO provides prolonged retinal tamponade and improves the success rate in these challenging cases, it is associated with a high incidence of complications, such as keratopathy, glaucoma, and cataract [3–6].

Glaucoma is one of the most common complications of SO which develops by a variety of pathophysiologic mechanisms including pupillary block, inflammation, synechial angle closure, rubeosis iridis, migration of emulsified, or nonemulsified SO into the anterior chamber. Infiltration and obstruction of the trabecular meshwork directly by emulsified SO are thought to be the driving force in the pathophysiology of SO related secondary open angle glaucoma (OAG) [4–8]. When a secondary glaucoma develops, it is reasonable to proceed with SO removal alone if the retina is completely attached. Even if, SO removal is an effective treatment for lowering intraocular pressure (IOP); it does not always provide IOP control. Success rates of medical treatment in controlling high IOP levels in SO related secondary glaucoma vary between 30% and 78% [9, 10]. While the medical antiglaucomatous treatments have high failure rates in the management of glaucoma secondary to SO, conventional glaucoma surgery has a limited success and carries high risk of complications such as hypotony and phthisis [11, 12].

If angle closure is not a contributing mechanism, selective laser trabeculoplasty (SLT) might be an option for lowering IOP. A great number of studies reported that SLT can be used as an adjunctive therapy to topical glaucoma drops with low complication rates in OAG [13, 14]. The aim of this study was to investigate the potential efficacy of SLT for lowering the intraocular pressure (IOP) in patients with OAG secondary to emulsified SO, in whom IOP could not be controlled with maximum antiglaucomatous medical treatment.

## 2. Methods

This prospective, interventional, single center study enrolled 11 eyes of 11 patients with OAG secondary to emulsified SO. The patients who had previously undergone SO removal were referred to SLT because of uncontrolled IOP with maximum tolerable antiglaucomatous medical treatment between December 2011 and June 2012. All patients had elevated IOP levels at the time of SO removal and were sustained for at least 3 months after removal. The protocol of the study adhered to the tenets of the Declaration of Helsinki. All patients provided informed consent before the procedure. Inclusion criteria were as follows: (1) IOP ≥ 21 mmHg on maximum antiglaucomatous medical treatment; (2) SO droplets in the anterior chamber or anterior chamber angle at the time of removal; (3) an attached retina after removal; (4) an open anterior chamber angle allowing laser application. Exclusion criteria were (1) any type of angle closure glaucoma; (2) an elevated IOP level attributed to previous vitreoretinal surgery other than emulsified SO, such as a scleral buckling procedure; (3) previous laser or surgical glaucoma interventions; (4) the diagnosis of glaucoma antedating vitreoretinal surgery.

Data regarding demographic information, visual acuity, number of glaucoma medications, underlying retinal pathologic findings that required vitreoretinal surgery with SO injection, vitreoretinal surgical procedure, number of vitreoretinal surgeries preceding SO removal, time of SO removal, time elapsed to the first observation of IOP elevation, and status of the lens and anterior chamber angle were recorded. Also parameters and complications of the SLT were recorded.

Preoperative evaluation including measurement of best corrected visual acuity via Snellen chart, measurement of IOP via Goldmann applanation tonometry, gonioscopy, slit lamp biomicroscopy, stereoscopic optic disc evaluation with a 90 diopter lens, and dilated fundus examination with binocular indirect ophthalmoscopy was performed.

### 2.1. Silicone Oil Removal Method

Silicone oil removal was performed via a pars plana entry. First, an infusion cannula was inserted into the vitreous cavity from an incision at the inferotemporal quadrant 3.0 mm away from the limbus in aphakic and pseudophakic eyes or 3.5 mm in phakic eyes. Then, another two pars plana incisions were made at the superotemporal and superonasal quadrants. One incision was used for illuminating the fundus to detect any redetachment at the time of the SO removal. SO was aspirated with a syringe by active aspiration. Then, anterior chamber was washed with a balanced salt solution (BSS Plus, Alcon Laboratories, Fort Worth, Texas, USA) to remove all SO droplets. Incision sites were closed with 7-0 vicryl suture.

### 2.2. Selective Laser Trabeculoplasty

Immediately before the laser procedure a single application of proparacaine hydrochloride 0.5% was instilled into the eye. All patients were treated by the same physician (BS). SLT consisted of Nd:YAG laser with a pulse duration of 3 ns and a spot size of 400 *μ*m was performed with the SLT Laserex Tango laser system (Laserex Tango, Ellex Medical, Australia). The energy level ranged from 0.6 to 1.1 mJ. The laser energy was initially set at 0.6 mJ until a cavitation bubble appeared. Then the laser energy was reduced by 0.1 mJ increments until no bubble formation was observed; treatment was then continued at this energy level. If no cavitation bubble was observed, the pulse energy was increased by increments of 0.1 mJ until bubble formation and then decreased as described above. The entire trabecular meshwork which could be seen easily and allowed to laser application was treated with contiguous laser spots. Apraclonidine eye drop was instilled once after SLT. No medical anti-inflammatory treatment applied and all patients continued with the same medical treatment after SLT. Postoperative examination including best corrected Snellen visual acuity, Goldmann applanation tonometry, slit-lamp biomicroscopy, and indirect ophthalmoscopy was performed at week 1, and at months 1, 3, and 6, postoperatively. Additionally, IOP in the treated eye was measured at first hour following SLT to detect IOP spike of ≥5 mmHg from baseline. If so, IOP spike was treated with appropriate antiglaucoma medications. The Wilcoxon signed-rank test was used for comparing pre- and post-SLT IOP measurements.

## 3. Results

Seven (63.6%) of the 11 patients were male and 4 (36.4%) were female. The mean age was 38.2 ± 16.6 years (range, 19–74 years). The pretreatment clinical characteristics of the patients are listed in Table 1.

The underlying pathology that required vitreoretinal surgery with SO injection was rhegmatogenous retinal detachment with proliferative vitreoretinopathy in 6 eyes, traumatic retinal detachment in 4 eyes, and proliferative diabetic retinopathy with tractional retinal detachment in one eye. All eyes had undergone pars plana vitrectomy with SO injection (1000-centistoke SO in 10 eyes, 5000-centistoke SO in 1 eye), additionally, 3 eyes scleral buckling with encircling, 1 eye lens extraction with phacoemulsification, and 2 eyes pars plana lensectomy at the time of pars plana vitrectomy with SO injection. Inferior peripheral iridectomies were performed at the time of pars plana vitrectomy with SO injection in aphakic eyes (2 eyes). All eyes had emulsified SO in the anterior chamber at the time of removal. After SO removal, gonioscopic examination revealed open drainage angle in 10 eyes and open drainage angle with 90° peripheral anterior synechia located around iridectomy site in one eye (Patient no. 8). We assume that peripheral anterior synechia might have been resulted from iridectomy procedure at the time of pars plana vitrectomy in patient 8. Furthermore, a few emulsified droplets of SO were identified at the superior anterior chamber angle without obscuring the angle structure on gonioscopy in all eyes (Table 2 summarizes patients' data).

**Figure 1:**
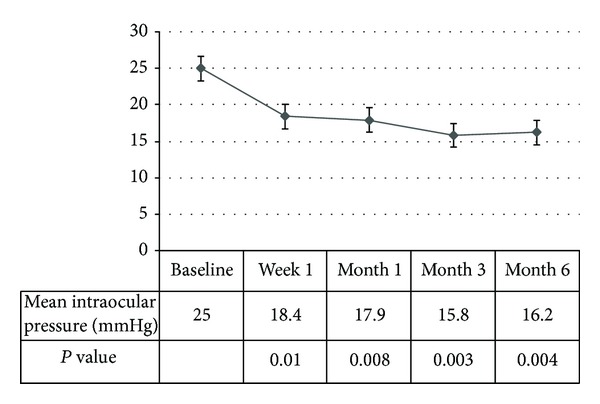
The course of mean IOP during the follow-up period.

The mean IOP was 25 ± 2.7 mmHg (range, 21–28 mmHg) at baseline. After SLT, the mean IOP decreased significantly to 18.4 ± 5.5 mmHg (range, 12–32 mmHg) at week 1, 17.9 ± 3.1 mmHg (range, 13–22 mmHg) at month 1, 15.8 ± 3.9 mmHg (range, 10–21 mmHg) at month 3, and 16.2 ± 4.7 mmHg (range, 11–24 mmHg) at month 6 (*P* = 0.01, 0.008, 0.003, and 0.004, resp.) (Figure 1). In one eye, IOP was measured as 32 mmHg at week 1 after SLT and returned to normal values with oral acetazolamide therapy for 3 days (Patient no. 5). IOP level of <21 mmHg at 6 months of therapy was achieved in ten eyes (91%). Pressure spike at the first hour following SLT was detected in one eye. Transient inflammatory reaction (1 + cells) was found in 2 eyes.

## 4. Discussion

Glaucoma is not a rare complication of retinal detachment surgery with the incidence of 6%–56%. Mechanisms of glaucoma with SO include pupillary block, inflammation, synechial angle closure, rubeosis iridis, migration of emulsified or nonemulsified SO into the anterior chamber, and idiopathic open-angle glaucoma [4–8]. Emulsification of SO carries a high risk of IOP elevation by mechanically obstructing the trabecular meshwork [15]. It has been confirmed by pathologic examination that emulsified SO drops or macrophages loaded with SO drops can block the trabecular meshwork. Elevated IOP is mainly dependent on the blockage of aqueous outflow [16]. Leaver et al. noted emulsified SO in 9 of 14 eyes showing elevated IOP after pars plana vitrectomy with SO injection. They reported histopathologic evidence of the presence of macrophages loaded with SO within the trabecular meshwork without evidence of structural damage to the collagen fibers and the trabecular endothelium [17]. In our study, the gonioscopic evaluation showed that the anterior chamber angle was open and no synechia was observed (except patient no. 8 with partial peripheral anterior synechia). We suppose that the elevated IOP mainly dependent on the blockage of outflow of aqueous humor by emulsified SO.

The benefit of early SO removal before the emulsification was demonstrated to be effective for IOP regulation in higher proportion of the eyes [18]. However, the removal of emulsified SO does not necessarily prevent the development of glaucoma. Flaxel et al. reported that elevated IOP persisted in all eyes with secondary glaucoma after SO removal [19]. Furthermore, SO removal itself can cause IOP elevation by splitting SO droplets into smaller bubbles which are more likely to obstruct trabecular meshwork during SO removal [20]. In the study by Wei et al., IOP was demonstrated to be elevated postoperatively in 11 of 64 cases after SO removal [21]. The incidence of glaucoma was reported to be 4.7% after SO removal in a study by Casswell and Gregor [22]. Lin et al. suggested that anterior chamber irrigation is important to remove all SO droplets to avoid IOP elevation secondary to emulsified SO [20]. Irrigation of anterior chamber as much as possible may be helpful to remove SO droplets; however, it is difficult to remove all retained SO droplets in the trabecular meshwork and to control the elevated IOP with this maneuver as in our patients. Moisseiev et al. performed anterior chamber wash-out for several times in an attempt to remove residual SO droplets and to achieve better IOP control after SO removal, but the method was not found to be effective in 91% of the eyes [18].

Treatment of patients with glaucoma secondary to SO is still controversial. In the case of glaucoma secondary to SO, aqueous suppressants should be the first line therapy to lower the pressure [23]. However, the ability to control IOP with glaucoma medications shows variable efficacy, and success rates vary between 30% and 78% [9, 10]. On the other hand, traditional filtering surgery is technically difficult because of the conjunctival scarring that results from the multiple retinal surgeries and carries a poor prognosis [9]. Glaucoma drainage implants may be another surgical option in the patients with glaucoma secondary to SO; however, there is a possibility of SO escape via the drainage tube [24]. Successful results with cyclodestructive procedures for lowering IOP have been reported in 74% to 82% of patients with medically uncontrolled glaucoma secondary to SO at one year. Unfortunately, multiple treatments may be required to obtain pressure control and visual loss is not uncommon after cyclophotocoagulation. For this reason, cyclodestructive procedures are less desirable treatment options in such patients [11, 25, 26].

Previous studies verified that SLT was safe and effective for lowering IOP in patients with OAG [13, 14, 27, 28]. SLT uses the 532 nm laser targeting the trabecular meshwork where the most resistance to aqueous outflow exists [27]. Several experimental studies using SLT have shown a release of chemotactic and vasoactive agents. These cytokines cause remodeling of the extracellular matrix and are involved in stimulation of cellular activity by increasing the recruitment and number of macrophages [27, 29]. We propose that activating the macrophages loaded with SO and remodeling the extracellular matrix in the trabecular meshwork by laser application may lead to increased aqueous outflow in glaucoma secondary to emulsified SO as in other types of OAGs. Latina and De Leon found that 40 eyes (64.5%) which were on maximum tolerable medical therapy achieved an IOP reduction of ≥20% from baseline with 360 degree SLT at month 12 [27]. Lowering the IOP < 21 mmHg was achieved in 91% of eyes without a significant complication in the present study. This difference in the success rates between the studies can be explained by the differences in success criteria, clinical characteristics of the patients, and duration of follow-up time.

A logical approach for the management of patients with glaucoma secondary to SO should be modified according to the individual clinical presentation. When emulsified SO blocks the trabecular meshwork, it is reasonable to proceed with SO removal and topical antiglaucomatous treatment, if the retina is completely attached. We suggest that SLT may be a treatment option for the patients with OAG secondary to emulsified SO which are not at high risk for progressive glaucomatous damage to save time before more invasive surgical interventions are performed.

The limitations of this study were the lack of a control group, limited number of the patients, and short follow-up period. Despite limitations, as a proof of principle pilot study, it may raise possibilities of further investigations on the role of IOP lowering potential of SLT in these challenging cases of glaucoma.

In conclusion, this study shows that SLT is a safe and effective method for lowering IOP in OAG secondary to emulsified SO. To our knowledge, this is the first report of safety and efficacy of SLT as an adjunctive therapy for emulsified SO related glaucoma. Although early results of this study are encouraging, long-term outcomes are needed to determine the efficacy of this therapy. Further investigations on a larger number of eyes are necessary to determine the efficacy of SLT in the patients who suffered from glaucoma secondary to emulsified SO.

## Figures and Tables

**Table 1:** Demographic and clinical characteristics and selective laser trabeculoplasty parameters.

Variables	Patients (*n* = 11)
Age (years), mean ± SD (range)	38.2 ± 16.6 (19–74)
Gender, male/female	7/4
Number of medications before SLT, mean ± SD (range)	2.9 ± 0.8 (2–4)
Pre-SLT Snellen BCVA (%)	
>20/200	1 (9%)
<20/200-CF	6 (54.6%)
≤HM	4 (36.4%)
PreSLT IOP (mmHg), mean ± SD (range)	25 ± 2.7 (21–28)
SLT parameters	
Number of laser spots, mean ± SD (range)	113 ± 45 (50–160)
Total energy (mJ), mean ± SD (range)	0.82 ± 0.16 (0.7–1.1)
Degree of angle treated, mean ± SD (range)	184 ± 43 (120–270)

SD: standard deviation; SLT: selective laser trabeculoplasty; BCVA: best corrected visual acuity; CF: counting fingers; HM: hand motion; IOP: intraocular pressure.

**Table 2:** Summary of patient data.

Patient number	Age	Sex	Underlying pathology	Latest VRS	Lens status	Time to SOR from VRS (mo)	Time to SLT from SOR (mo)	Angle status	Time to IOP elevation from VRS (mo)	Pre-SLT maximum IOP (mmHg)	Pre-VRS IOP (mmHg)	Previous surgeries
1	25	F	RD with PVR	PPV, SBE, SOI	Pseudophakic	16	28	Open	3	30	10	PE + PCL
2	74	F	RD with PVR	PPV, SBE, SOI	Pseudophakic	46	9	Open	40	35	16	PE + PCL
3	51	M	TRD	PPV, SOI	Pseudophakic	18	7	Open	5	24	14	PPV, PE + PCL
4	52	M	RD with PVR	PPV, SOI	Pseudophakic	14	46	Open	12	44	12	PE + PCL
5	31	M	TRD	PPV, PPL, SBE, PI, SOI	Aphakic	3	10	Open	2	22	17	—
6	19	M	RD with PVR	PPV, PPL, PI, SOI	Aphakic	8	11	Open	7	40	11	—
7	31	F	PDRP and RD	PPV, PE + PCL, SOI	Pseudophakic	7	54	Open	6	28	16	—
8	23	M	TRD	PPV, SOI	Pseudophakic	6	7	Open and 90° PAS	4	36	14	PPV, PI, ECCE + PCL
9	29	M	TRD	PPV, SOI	Pseudophakic	13	28	Open	3	40	14	PPV, PI, PE + PCL
10	51	F	RD with PVR	PPV, SOI	Pseudophakic	15	3	Open	4	42	11	PE + PCL
11	35	M	RD with PVR	PPV, SOI*	Pseudophakic	7	15	Open	4	38	10	PPV, SB, PE + PCL

VRS: vitreoretinal surgery; SOR: silicone oil removal; SLT: selective laser trabeculoplasty; IOP: intraocular pressure; RD: retinal detachment; PVR: proliferative vitreoretinopathy; PPV: pars plana vitrectomy; SBE: scleral buckling with encircling; SOI: silicone oil injection; PE: phacoemulsification; PCL: posterior chamber lens implantation, TRD: traumatic retinal detachment; PI: peripheral iridectomy; PPL: pars plana lensectomy; PDRP: proliferative diabetic retinopathy; ECCE: extracapsular cataract extraction; PAS: peripheral anterior synechia.

*5000-centistoke silicone oil.
